# Supplementary material for: Interventions to Improve Knowledge, Attitudes, and Uptake of Recommended Vaccines during Pregnancy and Postpartum: A Scoping Review
Source: Vaccines (Basel). 2023 Nov 21;11(12):1733. doi: 10.3390/vaccines11121733 (PMC10747157; doi:10.3390/vaccines11121733)
Supplement: Supplementary file 1 [file vaccines-11-01733-s001.zip › vaccines-2635140-supplementary.pdf]

Supplementary Table S1: PRISMA Extension for Scoping Reviews (PRISMA-ScR) checklist [24]

| SECTION | ITEM | PRISMA-ScR CHECKLIST ITEM | REPORTED ON PAGE # |
|---|---|---|---|
| **TITLE** | | | |
| Title | 1 | Identify the report as a scoping review. | 1 |
| **ABSTRACT** | | | |
| Structured summary | 2 | Provide a structured summary that includes (as applicable): background, objectives, eligibility criteria, sources of evidence, charting methods, results, and conclusions that relate to the review questions and objectives. | 1 |
| **INTRODUCTION** | | | |
| Rationale | 3 | Describe the rationale for the review in the context of what is already known. Explain why the review questions/objectives lend themselves to a scoping review approach. | 2 |
| Objectives | 4 | Provide an explicit statement of the questions and objectives being addressed with reference to their key elements (e.g., population or participants, concepts, and context) or other relevant key elements used to conceptualize the review questions and/or objectives. | 2 |
| **METHODS** | | | |
| Protocol and registration | 5 | Indicate whether a review protocol exists; state if and where it can be accessed (e.g., a Web address); and if available, provide registration information, including the registration number. | N/A |
| Eligibility criteria | 6 | Specify characteristics of the sources of evidence used as eligibility criteria (e.g., years considered, language, and publication status), and provide a rationale. | 3 |
| Information sources | 7 | Describe all information sources in the search (e.g., databases with dates of coverage and contact with authors to identify additional sources), as well as the date the most recent search was executed. | 3 |
| Search | 8 | Present the full electronic search strategy for at least 1 database, including any limits used, such that it could be repeated. | 1-2 (Table S2) |
| Selection of sources of evidence | 9 | State the process for selecting sources of evidence (i.e., screening and eligibility) included in the scoping review. | 3 |
| Data charting process | 10 | Describe the methods of charting data from the included sources of evidence (e.g., calibrated forms or forms that have been tested by the team before their use, and whether data charting was done independently or in duplicate) and any processes for obtaining and confirming data from investigators. | 3-4 |
| Data items | 11 | List and define all variables for which data were sought and any assumptions and simplifications made. | 3-4 |
| Critical appraisal of individual sources of evidence | 12 | If done, provide a rationale for conducting a critical appraisal of included sources of evidence; describe the methods used and how this information was used in any data synthesis (if appropriate). | N/A |
| Synthesis of results | 13 | Describe the methods of handling and summarizing the data that were charted. | 4-12 (Table1, Table S3) |
| **RESULTS** | | | |
| Selection of sources of evidence | 14 | Give numbers of sources of evidence screened, assessed for eligibility, and included in the review, with reasons for exclusions at each stage, ideally using a flow diagram. | 3 Figure 1 |
| Characteristics of sources of evidence | 15 | For each source of evidence, present characteristics for which data were charted and provide the citations. | 6-17 (Table 1, Table S3, Figure 2 and 3) |
| Critical appraisal within sources of evidence | 16 | If done, present data on critical appraisal of included sources of evidence (see item 12). | N/A |
| Results of individual sources of evidence | 17 | For each included source of evidence, present the relevant data that were charted that relate to the review questions and objectives. | 6-17 |
| Synthesis of results | 18 | Summarize and/or present the charting results as they relate to the review questions and objectives. | 6-17 |

| SECTION | ITEM | PRISMA-ScR CHECKLIST ITEM | REPORTED ON PAGE # |
|---|---|---|---|
| **DISCUSSION** | | | |
| Summary of evidence | 19 | Summarize the main results (including an overview of concepts, themes, and types of evidence available), link to the review questions and objectives, and consider the relevance to key groups. | 6-19 (Table 1, Table S3, Figure 2 and 3) |
| Limitations | 20 | Discuss the limitations of the scoping review process. | 19 |
| Conclusions | 21 | Provide a general interpretation of the results with respect to the review questions and objectives, as well as potential implications and/or next steps. | 19 |
| **FUNDING** | | | |
| Funding | 22 | Describe sources of funding for the included sources of evidence, as well as sources of funding for the scoping review. Describe the role of the funders of the scoping review. | 19 |

Supplementary Table S2: Search strategies used in this scoping review:

| **Database** | **Search strategy** |
|---|---|
| PubMed | ""pregnant women""[MeSH Terms] OR ""pregnancy""[MeSH Terms] OR ""postpartum period""[MeSH Terms] OR ""postpartum women""[Title/Abstract] OR ""expecting mother""[Title/Abstract]) AND (""vaccine acceptance""[Title/Abstract] OR ""vaccine uptake""[Title/Abstract] OR ""vaccine confidence""[Title/Abstract] OR ""vaccine hesitancy""[Title/Abstract] OR ""vaccine intention""[Title/Abstract]) AND (""early intervention, educational""[MeSH Terms] OR ""intervention""[Title/Abstract] OR ""educational intervention""[Title/Abstract])" |
| Scopus | ( "intervention" OR "educational intervention" ) AND ( "vaccine acceptance" OR "vaccine uptake" OR "vaccine intention" OR "vaccine hesitancy" ) AND ( "pregnant women" OR "postpartum women" OR "expecting mother" ) AND ( LIMIT-TO ( DOCTYPE , "ar" ) ) AND ( LIMIT-TO ( LANGUAGE , "English" ) OR LIMIT-TO ( LANGUAGE , "French" ) ) AND ( LIMIT-TO ( SUBJAREA , "MEDI" ) OR LIMIT-TO ( SUBJAREA , "SOCI" ) OR LIMIT-TO ( SUBJAREA , "MULT" ) OR LIMIT-TO ( SUBJAREA , "NURS" ) OR LIMIT-TO ( SUBJAREA , "PSYC" ) ) AND ( LIMIT-TO ( SRCTYPE , "j" ) ) |
| Web of Science | ((ALL=("pregnant women" OR "pregnancy" OR "expecting mother" OR "postpartum women" OR "antenatal" OR "prenatal" )) AND ALL=("Vaccine acceptance" OR "Vaccine uptake" OR "Vaccine intention" OR "vaccine hesitancy")) AND ALL=("intervention" OR "educational intervention") |
| EBSCOHost | S2: pregnant women or pregnant women or pregnancy or pregnant or expecting mother<br>S3: antenatal or prenatal or pre-birth or pregnancy<br>S4: vaccine acceptance or vaccine hesitancy or anti-vax or anti-vaccination or immunization<br>S5: vaccine uptake or vaccination or immunization or vaccines<br>S6: interventions or strategies or best practices |

| | |
|---|---|
| | S7: S2 OR S3<br>S8: S4 OR S5<br>S9: S6 AND S7 AND S8 |
| Google Scholar | "Vaccine uptake" "vaccine acceptance" "vaccine hesitancy" "pregnant women" "postpartum women |

Supplementary Table S3: Characteristics of the studies included in this scoping review

| Author (Publication year) | Country | Vaccine (s) | Study design and sample size | Intervention (s) | Main reported findings |
|---|---|---|---|---|---|
| Paula M. Frewa et al, (2016), [27] | United States | Influenza | Randomized controlled trial. Sample n=95 | The aim of this 3-arm interventional study was to test 2 forms of targeted persuasive messaging models in comparison to generic influenza Vaccine Information Statements (VIS) developed by the CDC. 34 participants were randomly assigned to the control group (VIS), 31 to Arm 2 affective messaging intervention (“Pregnant Pause” video) and 30 to Arm 3 cognitive messaging intervention (“Vaccines for a Healthy Pregnancy” video). | At baseline, most women reported no receipt of seasonal influenza immunization during the previous 5 years. They showed low intention to vaccinate against influenza during their current pregnancy. At30-days postpartum follow-up, influenza vaccination uptake was low among all participants. Therefore, there was no effect after a single exposure to either affective messaging or cognitive messaging interventions on the vaccine uptake.<br>Authors concluded that, to enhance influenza coverage among pregnant women, it may be necessary for this population to get repeated influenza vaccination exposures with revisions to the message's format, contents, and relevance. |
| Valerie Wing Yu Wong et al, (2016), [28] | Hong Kong | Influenza | Randomized controlled trial. Sample n=305. | The objective of this intervention was to evaluate the efficacy of a brief, one-to-one education session on the influenza vaccination uptake during pregnancy and the proportion of participants seeking out influenza vaccination. Participants recruited from 4 antenatal clinics were randomized to receive either standard antenatal care or brief one-to-one education. Participants were follow-up via telephone call at 2 weeks postpartum. | The uptake was higher among participants who received brief education compared to the standard care group. More participants in the education group-initiated discussion about influenza vaccination with their health care provider, but the difference was not statistically significant. Of those who did not receive the influenza vaccine (n = 271), 45 attempted to get vaccinated. A significantly higher proportion of participants who attempted to get vaccinated were in the intervention group. Thus, in case participants who had attempted vaccination had received the vaccine, vaccination uptake rates would have been substantially higher in the intervention group. Authors concluded that even though the brief education was effective |

| | | | | | |
|---|---|---|---|---|---|
| | | | | | in improving vaccination uptake among pregnant women, overall vaccination rates remain suboptimal. Therefore, multicomponent interventions, including positive recommendations by healthcare professionals, are needed to promote maternal influenza vaccination uptake. |
| Yinan Li et al, (2022), [51] | Canada | Tetanus–diphtheria–acellular pertussis (Tdap) | Quasi experimental, multicentre study. Sample n= 946. | The aim of the intervention was to assess and compare the vaccine coverage associated with four province-based implementation models of maternal Tdap vaccine delivery which are: 1- the existing standard of practice model which is local community service centres (**CLSCs**), 2- family medicine groups (**FMGs**), 3- **obstetrics** clinic and, 4- the oral glucose challenge test (**OGCT**). | The highest model-specific vaccine coverage was achieved via FMGs, but coverage was not significantly different from the CLSCs. Compared with CLSCs, overall vaccine coverage was significantly higher when Tdap was offered in FMGs or an obstetrics clinic providing antenatal care, however, the OGCT model did not improve overall vaccine coverage. Health care providers who are involved in following up pregnant women recommending and offering the vaccine may be essential in improving vaccine coverage. |
| Claudio Costantino et al, (2021), [38] | Italy | Influenza and Tdap | Multicentre, pre-post educational intervention. Sample n=326. | A before and after educational intervention focused on vaccination during pregnancy, immunization during life-course and vaccination recommended in Italy was conducted by healthcare workers during the childbirth classes. | The educational intervention and the counselling by healthcare professionals, on vaccination during pregnancy during childbirth courses improved considerably the vaccination uptake during pregnancy. |
| Sara E Mazzoni et al, (2016), [50] | United States | Influenza, Tdap and Human papillomavirus (HPV) | Multicentre, pre-post multiples interventions. Sample n= 19,409 for Influenza, 2741 for Tdap and 12,443 for HPV. | Most interventions were aimed at enhancing standard processes for all vaccinations in the clinics and were adopted from evidence-based practices proven to improve vaccination coverage. Most of the implemented interventions in the current study were as follows: 1-Education sessions for non-provider medical staff were conducted to raise awareness and empower them in their leading role in provision of vaccinations. All medical staff at all clinics were required to attend 2 sessions, one on HPV and the other on Tdap in pregnancy. 2- The existing standing orders for vaccines were revised or expanded depending on the vaccine. 3- Prior to the intervention, a standing order would be provided only for a vaccine covered by insurance. This was revised to provide an order for all indicated vaccines regardless of insurance. The standing orders were also expanded to include influenza in | The uptake rate of influenza vaccination increased from 35.4% in the pre-intervention period to 46.0% after the intervention. After controlling for age, race/ethnicity, and insurance in GEE regression models for Tdap and HPV, patients remained significantly more likely to have been vaccinated during the intervention period than before. The overall raw average rate for up-to-date Tdap vaccination increased from 87.6% before the intervention period to 94.5% in the period after intervention.<br>Authors concluded that using evidence-based strategies largely established in other |

| | | | | | |
|---|---|---|---|---|---|
| | | | | the outpatient setting, as before the intervention there was only a standing order for influenza vaccine on the inpatient wards. A registered nurse who performed periodic chart reviews and gave immediate feedback to providers when missed opportunities were identified. Practices were different with regards to Tdap required additional interventions. Firstly, each clinic began stocking and administering Tdap, as this was the only of the 3 vaccines not available in ob-gyn clinics prior to the intervention. Additional staff trainings including providers were conducted with each recommendation change. Patient handouts were created and routinely given out at each first antenatal session and at ultrasound visits. Each obstetrics patient also has a paper chart, and the antenatal paper chart forms were revised to include a standing order for Tdap. The revised form also provided a space for documentation of vaccine discussions and enforced the intervention requirement of 3 documented Tdap and/or influenza vaccine refusals among patients who declined vaccination. | settings, interventions implemented in this study was associated with increased rates of influenza, Tdap, and HPV vaccination uptake in outpatient underserved obstetrics and gynecology clinics. Therefore, the integration of such evidence-based practices into routine obstetrics and gynecology care could positively impact preventive health for many women. |
| Joanne Parsons et al, (2022),[39] | United Kingdom | Influenza | Before and after interventional study. Sample n= 411. | The digital intervention consists of an approximately 4 minutes long animation and was informed by Intervention Mapping [71] which ensured that the intervention was based on theory and evidence, as well as being grounded in the needs and preferences of the target population. It addressed beliefs about the risk of Influenza and the efficacy of the vaccination, using animated messages to convey information to pregnant women. The animation informed pregnant women about the risks of Influenza to themselves and their unborn baby. The intervention content was specifically designed to provide explanations, and simple visual demonstrations of the processes involved in the infection by the Influenza virus in pregnant women, and how vaccination against Influenza works to disrupt it. The intervention aimed to increase vaccination uptake through changing pregnant women's risk and efficacy appraisals. Prior to receiving the intervention (baseline), and immediately afterward (follow-up one), participants were asked to complete a short questionnaire to measure risk appraisals. Approximately 6 months later, a further short questionnaire (follow-up two) was administered to measure vaccination behavior. Single- | The digital intervention implemented in the current study resulted in an increased appraisal of likelihood of getting flu during pregnancy and severity of Influenza infection during pregnancy, and increased intentions to accept Influenza vaccination during pregnancy. Of the 67 respondents who completed follow-up survey two, 38 reported having the vaccination while pregnant. Findings from this study showed evidence supporting the promise of the implemented intervention. Randomized controlled trials are required to produce definitive efficacy evidence. |

| | | | | | |
|---|---|---|---|---|---|
| | | | | item measures of study variables (severity, likelihood, and vaccination intentions and behavior) were employed to keep the survey short to maximize recruitment and retention. The questionnaires were delivered via Qualtrics survey software. | |
| Sean T. O'Leary et al, (2019), [53] | United States | Influenza, Tdap and HPV | A 2- arm cluster randomized controlled trial including 9 private ob-gyn practices.<br>Sample n= 32,590 per arm. | The intervention consisted of designation of vaccination champions, staff/provider trainings, assistance with vaccine purchasing/management, identification of eligible patients, standing order implementation, chart review/feedback, and patient education materials. Control practices continued usual care. | There were not significant differences between intervention and control groups for uptake of influenza vaccine among pregnant women, with both study arms increasing their uptake. Results from this study showed an increase of 2% in the intervention arm and 11% in the control arm. In addition, there were not significant differences in uptake of Tdap vaccine in the intervention group compared to the control group. In the Baseline Period, 18% of pregnant women in the intervention arm received Tdap vaccine versus 22% in the control arm. Besides, given the change in recommendations during the study period, these proportions increased dramatically in both arms during year three of the study period. Both intervention and control groups increased to 51% in the Post-Intervention Period, showing an increase of 33% for the intervention arm and 29% for the control arm. In this cluster randomized trial designed to increase vaccination uptake, both intervention and control practices showed improved vaccination of pregnant even though the differences between both groups were not statistically significant. Future work should focus on tailoring evidence-based vaccination interventions or developing new strategies to specifically fit busy obstetrics and gynecology clinics . |
| Sylvia Yeh et al, (2014), [48] | United States | Tdap | A 2- arm cluster randomized controlled trial including 2 hospitals.<br>Sample, n= 600 reviewed charts per hospital. | This was a 2-stage intervention: firstly, an "opt-in" order as part of the pre-printed postpartum orders was implemented at the end of November 2009. This required providers to check the order for both vaccinations to be given to women after delivery before hospital discharge. Then the intervention simplified the delivery of vaccinations by implementing a | Tdap postpartum vaccination uptake was 0% at both hospitals at prior the start of the intervention. In the intervention hospital, the introduction of the opt-in order was followed by an increase in postpartum vaccination to 18%. The introduction of the standing order |

| | | | | | |
|---|---|---|---|---|---|
| | | | | policy with standing orders for postpartum vaccination for Tdap and seasonal and H1N1 influenza vaccination was implemented in February 2010. The standing orders empowered nurses to deliver influenza and/or Tdap vaccines without an additional order from the physician. Tdap would be administered unless the patient refused or had a contraindication to vaccination. The control hospital maintained standard practice. Randomly selected hospital charts of women after delivery were reviewed for receipt of Tdap and demographic data. Tdap vaccination rates among postpartum women were evaluated and review of 1252 charts was conducted (648 intervention hospital; 605 control hospital) from women with completed deliveries. | approach was resulted in a further increase to 69%. No postpartum Tdap vaccinations were documented in the comparison hospital. Postpartum Tdap vaccination in the intervention hospital did not differ by demographic characteristics.<br>Authors concluded that in-hospital ordering procedures substantially increased Tdap vaccination coverage in postpartum women. Opt-in orders increased uptake rate that increased substantially with standing orders. |
| Ya-Wen Chang et al, (2022), [37] | Taiwan | Influenza | Multicentre randomized controlled trial.<br>Sample n= 243. | The intervention involved an "Influenza Vaccination Reminder Application" and evaluated its efficacy in improving vaccination intention among pregnant women. Pregnant women were randomly assigned to the intervention group, to whom the "Influenza Vaccination Reminder Application" was provided for at least two months, while pregnant women<br>in the control group received standard care. | The intervention increased pregnant women's knowledge about influenza and vaccines, strengthened their positive attitudes towards maternal influenza vaccination, and promoted positive behavioral intention toward influenza vaccination and the difference between the experimental and control group was statistically significant. |
| Sean T. O'Leary, (2019), [29] | United States | Tdap and Influenza | Single centre randomized controlled trial (RCT).<br>Sample n= 289 for Influenza and 173 for Tdap. | This was a 3-arm RCT, women were randomly assigned to 1 of 3 arms: "website with vaccine information and interactive social media components", "website with vaccine information only", or usual care. Participants in the "website with vaccine information and interactive social media components" and "website with information about vaccination only" groups had access to the same base vaccine content. The "website with vaccine information and interactive social media components" included a blog, discussion forum, and "Ask a Question" portal as well. | Participants in both the "website with vaccine information and interactive social media components" and "website with vaccine information only" had higher vaccine uptake than the usual care group for the Influenza vaccine. There were no significant differences in vaccine uptake between study groups for the Tdap vaccine.<br>The current study demonstrated the Web-based interventions, with and without social media components, showed higher uptake of influenza vaccine in pregnant women receiving the intervention. Further studies are needed to determine the optimal timing and populations for administration of online interventions to increase Tdap and influenza vaccine uptake in pregnancy. |

| | | | | | |
|---|---|---|---|---|---|
| C. Bonneau et al, (2010), [47] | France | Tdap | Pre-post intervention study. Sample n=61. | The intervention was conducted by doctors and midwifes who provide oral (twice: midwife and doctor) and written information about pertussis and prescription for Tdap vaccine for postpartum women. | At follow-up, the vaccination uptake was 16%, including 8% related to the intervention (vaccinated during postpartum). Findings from the current study showed a limited effect of the implemented intervention. Authors agreed that Tdap vaccination among pregnant and postpartum women should also involve doctors before, during and after pregnancy, and pediatricians as well. |
| Stefania Bruno et al, (2021), [41] | Italy | Influenza | Pre-post intervention study. Sample n= 119 | The intervention consists of a vaccination training session was carried out during the birthing preparation course, aimed at increasing the attitude toward vaccination in pregnant women. A survey on vaccination awareness was administered before and after the training session and on-site Influenza vaccination was offered to women and their companions. | The percentage of participants who consider the preparatory course a useful tool to obtain information about vaccines increases significantly from 30.34% at pre-intervention to 64.56% at post-intervention. There was a significant increase in the mean number of vaccinations that the participants want their children to get. The number of participants believing that there is no relationship between vaccination and autism rose from 41.05 to 72.97%. In total, 48 out of 119 (40.34%) pregnant women were vaccinated against Influenza. Vaccination knowledge and attitude significantly increased after a training session dedicated to vaccination as a part of the pregnant pre-birth course. Authors concluded that the on-site vaccination educational sessions appear to be a useful public health tool to enhance the vaccination compliance and reduce the supply-side vaccination barriers. |
| Kenneth Goodman et al, (2015), [30] | United States | Influenza | RCT. Sample n=105. | Unvaccinated patients seen for routine antenatal care were randomized to pre-visit vaccination video education or control. Pre and post video health beliefs were assessed and unvaccinated women were subsequently interviewed by phone. The intervention consisted of an educational video that addresses vaccination health beliefs concepts found to be predictive of vaccination and is intended to contain a clear and | In 105 randomized participants, intervention positively influenced health beliefs as demonstrated by differences in mean pre- vs. post scores for intervention vs. control. Vaccination uptake rates were 28% intervention and 25% control. Provider recommendation was associated with vaccination (47% if recommended vs.12% if not). Phone interviews showed susceptibility |

| | | | | | |
|---|---|---|---|---|---|
| | | | | easy to understand format. Those in the control group viewed another video addressing handwashing hygiene. | to influenza and vaccine safety as primary reasons not vaccinating. Authors agreed that the video education positively influenced vaccination health beliefs without impacting vaccination uptake rates. Physician's recommendation was strongly associated with participant's decision to vaccinate and may be most effective when emphasizing influenza vaccination's protective impact on the newborn. |
| A.T. Chamberlaina et al, (2015), [54] | United States | Influenza and Tdap | A cluster-randomized trial. Sample n=325. | The intervention was multi-component and involved identification of a vaccine champion, provider-to-patient talking points, educational brochures, posters, lapel buttons, and iPads loaded with a patient-centred tutorial. Control hospitals were asked to maintain their standard care practice. | Despite antenatal influenza and Tdap vaccination uptake were higher in the intervention group than the control group, improvements were not statistically significant. While most of the intervention package components were positively associated with prenatal vaccine receipt, a provider’s recommendation was the factor most strongly associated with actual receipt, regardless of study group or vaccine. |
| Michelle H. Moniz et al, (2013), [31] | Canada | Influenza | Single centre RCT. Sample n=216. | In both groups participants received 12 weekly text messages. The control group received text messages regarding general preventive information regarding pregnancy; and the intervention group received text messages regarding general preventive health information in pregnancy plus the importance of influenza vaccination during pregnancy. | The overall influenza vaccination uptake rate among participants was 32% with no difference between participants in the control (31%) compared with to the intervention group (33%). In conclusion, text messaging intervention was not effective at increasing influenza vaccination uptake rates among a low-income, urban, ambulatory pregnant population. |
| Po-Jen Cheng et al, (2015), [52] | Taiwan | Tdap | Pre-post intervention study. Sample n= total of 7785 postpartum women were evaluated, 4599 from the pre-intervention and 3186 from the post-intervention. | The intervention included intensive physician and nursing education programs to ensure that all obstetric physicians, clinic nurses and medical assistants had basic knowledge of early-onset neonatal Streptococcus Group B (GBS) infection, neonatal pertussis infection, perinatal preventive strategies for both GBS and pertussis. Then, an office-based intervention was set up and incorporated pertussis education program into prenatal GBS screening clinics. During the antenatal care clinic and when pregnant women came for GBS screening, all of them received an education sheet including the message | Tdap vaccination was more likely during the post-intervention period compared with the pre-intervention period. Comparisons between each subgroup of pre-intervention and post-intervention women demonstrated that incorporating pertussis information into prenatal education for GBS prevention was beneficial except for women of maternal age 30–34 years and women living in rural areas. Prenatal GBS screening sessions represent an |

| | | | | | |
|---|---|---|---|---|---|
| | | | | "Neonatal Pertussis is Preventable! Neonatal GBS is Preventable!". After GBS screening was conducted, healthcare providers educated.<br>all pregnant women about the importance of GBS screening, and postpartum Tdap immunization for neonatal infectious protection. Lastly, a prenatal Tdap education alert was incorporated into the electronic prenatal care medical record system and consisted of an electronic reminder. In case a physician ordered culture of vaginal and rectal swab cultures for possible GBS colonization for a woman in the target population (women without Tdap vaccination documentation) the antenatal Tdap education alert reminded healthcare providers with the following message: "Health education about Tdap vaccination recommended for postpartum women." | opportunity for healthcare providers to offer pertussis postpartum vaccination information to eligible pregnant women to improve the uptake of Tdap vaccination among postpartum women. |
| Joseph G. Giduthurim et al, (2019), [46] | India | Influenza | Multicentre RCT.<br>Sample n= 30 clinicians. | Clinicians were randomized to an intervention and control arm in slum and middle-class study sites. The study commenced in July 2015, lasted for 11 months, and included 2 main activities with clinicians in the intervention group. First, clinicians were interviewed and provided with Antenatal Influenza Vaccination (AIV) implementation recommendations (global, academic, and local) aimed to motivate clinicians' influenza vaccination practices for pregnant women coming for antenatal care (ANC). Then, a qualitative community survey of women and some of their spouses was performed in neighbouring communities of active clinics to assess community views about vaccination, and particularly for AIV during pregnancy. Findings from the survey were summarized and briefly presented to clinicians in during the second activity. Lastly, at all participating study clinics, both active and control clinics, influenza and tetanus vaccination status was noted in all daily ANC-visit records during the entire study period. The status of tetanus toxoid (TT) vaccination was monitored as an indication of the clinic capacity to vaccinate women receiving ANC. | After first and second intervention activities, clinicians in the intervention arm middle-class communities vaccinated at 12.2% and 37.8%, respectively. Middle-class clinicians in the control group vaccinated at <0.2% throughout the study. This difference in AIV taken opportunities between middle-class active and control clinics was statistically significant after first and second intervention activities. In slum-community intervention sites, clinician AIV activity was minimal throughout the study period.<br>Authors conclude that their approach for engaging clinicians effectively reduced missed opportunities for AIV in urban middle-class settings. It may also improve maternal vaccination for other conditions.<br>The absence of any similar impact in slum-based clinics might be the result of critical limitations of vaccine access. |
| Melissa S. Stockwell et al, (2014), [32] | United States | Influenza | Multicentre RCT.<br>Sample n= 1187. | Participants in the intervention group received five weekly text messages regarding influenza vaccination starting mid-September 2011 and 2 text message appointment reminders. Participants in both groups received standard automated | Participants in the intervention arm were 30% more likely to receive the vaccine after adjusting for gestational age and number of clinic visits. The sub-group analysis revealed that participants early in their third trimester |

| | | | | | |
|---|---|---|---|---|---|
| | | | | telephone appointment reminders. Primary outcomes were receipt of either prenatal or postpartum influenza vaccination. | at randomization showed the greatest intervention effect. Findings from this interventional study showed that text messaging was associated with increased influenza uptake, in a low-income obstetric population, mainly those who received the intervention early in their third trimester. |
| Helena C. Maltezou et al, (2019), [40] | Greece | Influenza | Before and after interventional study. Sample n= 304. | The interventional study consisted of an educational that included a leaflet that was given if the participant asked for it. It included information about the complications due to influenza infection among pregnant women and neonates and the efficacy and safety of influenza vaccine administered during pregnancy. The leaflet was provided to pregnant women at the Outpatient Clinic while waiting for their appointment. The potential benefits of influenza vaccination in pregnancy were also presented by their obstetrician. Participants also discussed with their obstetrician their concerns about vaccination. | The educational intervention was associated with an increased influenza vaccination uptake rate of 19.5% among pregnant women compared to <2% the past years. Authors agreed that more rigorous interventions should be conducted to enhance vaccine uptake by pregnant women. |
| Pamela M. Meharry et al, (2013), [33] | United States | Influenza | Multicentre RCT. Sample n=135 | The intervention included a pamphlet that was tailored to pregnant women entitled "Influenza in Pregnancy", with the subtitle, "One Flu Shot Protects You and Your Baby". The content covered various sections on what pregnant women should know about influenza and the vaccination to prevent illness. The participants were randomized to one of three groups: the pamphlet group only; pamphlet plus benefit statement "vaccinating the pregnant woman also benefits the young infant"; or control. | Overall, 66.9 % of the participants received the Influenza vaccine. Both the pamphlet group (72.9 %) and the pamphlet plus benefit statement group (86.1 %), had significantly higher vaccination uptake rate than the control group (46.9 %). The pamphlet significantly increased the pregnant women's perceptions of the safety, benefits of vaccination against Influenza during pregnancy, and the overall uptake. |
| Matthew Z. Dudley et al, (2022), [34] | United States | Influenza | Multicentre RCT. Sample n= 2087. | MomsTalkShots was the patient-level component of a multi-level intervention to enhance vaccination uptake during pregnancy and childhood. The intervention also included provider- and practice-level interventions. All participants (n=2087) received a baseline questionnaire of maternal and childhood vaccine attitudes, and 2 follow-up surveys. Half of participants (n=1041) were randomly assigned to receive educational videos through MomsTalkShots, algorithmically tailored to their vaccine attitudes including intentions, and demographics. Because the practice/provider intervention did not seem to be effective, this analysis focused on | At 1-month postpartum follow-up, MomsTalkShots increased perceived risk of maternal Influenza infection among participants in the intervention group, of Influenza vaccine efficacy, and perceived vaccination knowledge. Among participants who were intending not to vaccinate at baseline, MomsTalkShots increased perceived risk of maternal Influenza infection and confidence in Influenza vaccine efficacy. Authors concluded that the intervention based on the MomsTalkShots application |

| | | | | | |
|---|---|---|---|---|---|
| | | | | MomsTalkShots regardless of the practice/provider intervention. | improved pregnant women's knowledge and perceptions of maternal vaccination and vaccines preventable diseases. |
| Saad B. Omer et al, (2022), [55] | United States | Influenza and Tdap | Cluster- and individually randomized controlled trial.<br>Sample n= 2092. | The intervention consisted of a multi-level, cluster- and individually randomized controlled trial. The intervention targeted practice-, provider- and patient-level barriers to vaccine uptake. Obstetric clinics were randomized to receive the practice and provider-level interventions or continue their usual standard of care. Participants were enrolled at each clinic and randomized women into patient-level intervention and control groups, resulting in four study arms. The practice level intervention consisted of the identification of a vaccination champion and the implementation of the Assessment, Feedback, Incentives and Exchange (AFIX) program [56]. Provider-level interventions included Continuing Medical Education (CME) module: based on five behavioural constructs and approaches. A copy of A Clinician's Guide to Vaccine Safety was provided at each clinic. For the patient-level intervention component a theory-driven individually tailored application called MomsTalkShots was developed. All participants were given a tablet and asked to complete a short survey capturing patient-level sociodemographic characteristics and vaccine attitudes, beliefs, and intentions. Women in the patient-level intervention arm then received video messages tailored to address the identified knowledge gaps and concerns and baseline vaccine intent based on responses to specific questions in the baseline survey. Women could receive up to six videos depending on their specific concerns. Women randomized to the control arm completed the baseline survey but did not receive any videos. | There was no statistically significant increase in Tdap or influenza vaccine uptake overall. Among women who had no intention of or were unsure about receiving the influenza vaccine while they are pregnant, those who received just the patient-level intervention were 61% more likely to receive the influenza vaccine than those in the control group.<br>There was no statistically significant difference in vaccine uptake for either influenza or Tdap between the four study arms. |
| Nutan B. Hebballi et al, (2022), [42] | United States | Tdap | Before and after intervention study.<br>Sample n=200. | A brief educational intervention session about maternal pertussis and Tdap vaccine was given to interested hospitalized postpartum women after which the Tdap vaccine was offered to eligible patients who did not receive it while they were pregnant. Medical records were reviewed to determine if surveyed participants received the vaccine prior to discharge. | 103 (51.5%) of the participants had received Tdap vaccinations prior to hospitalization. Of those 97 who were not vaccinated with Tdap prior to admission but were eligible to receive vaccine, 24 (25%) were vaccinated prior to study as part of routine hospital-based screening and vaccination program, 29 (38.2%) after |

| | | | | | |
|---|---|---|---|---|---|
| | | | | | our survey. Authors concluded that educational interventions to targeting pregnant patients about the advantages of vaccination for their unborn child, addressing patient safety concerns, and vaccine administration in obstetricians' practices may significantly improve maternal vaccination uptake rates. |
| Paula M. Frewa et al, (2014), [35] | United States | Influenza | Single centre 3-arm RCT. Sample n= 272. | The intervention consisted of a randomized exposure to 2 types of messages: Positively oriented ("gain-frame") messages communicate information by emphasizing the benefits of receiving the vaccine; and negatively oriented ("loss-frame") messages emphasize the risks of not receiving the vaccine. Participants were randomized to receive one of three types of vaccine messages standard. vaccine information sheet (control), gain-frame, or loss-frame messages. | Neither gain- nor loss-framed messages were significantly associated with increased likelihood of influenza vaccination among pregnant women. |
| Mark H. Yudin et al, (2017), [36] | Canada | Influenza | Single centre RCT. Sample n=317. | The intervention included text message reminders and aimed to evaluate if it will increase the likelihood of getting vaccinated against Influenza among pregnant women. Participants were informed that the messages would be about health-related behaviour in pregnancy. Those randomized to the intervention arm received two messages weekly for four consecutive weeks reinforcing that vaccination against Influenza is recommended for all pregnant women and safe during pregnancy and breastfeeding. | There were 31% participants in the intervention group and 27% participants in the control group received the vaccine. Therefore, weekly text messages emphasising the recommendation for and safety of the influenza vaccine in pregnancy did not increase the likelihood of getting vaccinated during pregnancy. Authors highlighted that overall vaccination uptake rates were low, pointing out the need for patient education and innovative strategies to enhance vaccine acceptance. |
| Aaliyah Momani et al, (2023), [43] | Jordan | COVID-19 | Multicentre quasi-experimental pre-post intervention study. Sample n=436. | The intervention group targeted women planning for pregnancy, pregnant women, and finally, women who are breastfeeding and more specifically, those who are not vaccinated against COVID-19. The intervention involved an individual-centred tele-education (interactive education phone sessions, phone calls consultancy, text messages, and digital education booklet) given to women in the intervention group for a duration of 2 weeks. The researcher invited participants of the intervention group and discussed COVID-19 disease, the benefits of the vaccine, effectiveness, side effects of the avail- | Participants in the intervention group reported significantly higher COVID-19 vaccine uptake rate and lower mean score of hesitancy than the control group. In addition to that, prior the implementation of the intervention, women in the intervention group reported significantly higher levels of hesitancy compared to those in the same group after the intervention. Authors concluded that education of pregnant women after being provided with the tele-education intervention about |

| | | | | | |
|---|---|---|---|---|---|
| | | | | able vaccines, sources of their information about the vaccine, and answered their questions related the disease and the vaccination. Adding to that, women had the chance to contact the researcher asking for information/further explanation over the phone. No intervention was administered to the control group. 62.9% of the participants in this study were pregnant (46,8%) or breastfeeding women (16.1%). | COVID-19 vaccination decreased their hesitancy and improved their willingness to be vaccinated against COVID-19. |
| William E. Trick, et al, (2010), [49] | United States | Tdap | Before and after intervention study. Sample n=431, 183 from the pre-intervention and 248 from the post-intervention periods. | The intervention targeted postpartum women and consisted of a computer-based clinical decision support system which is an application incorporated into the hospital's information system; the system uses information in the electronic medical record to provide logic-based diagnostic or therapeutic guidance to clinicians. When a physician entered an order for iron supplementation, a dialogue box was displayed that contained a preselected order for Tdap vaccine that read "Tdap vaccination is recommended for postpartum mothers. An order for Tdap will be generated and sent to Pharmacy and Nursing unless you deselect the order below. The frequency of Tdap vaccination uptake during the pre-intervention and post-intervention periods was compared. | Tdap vaccine uptake was more likely for postpartum women during post-intervention compared to the pre-intervention time periods (59% compared to 0% during pre-intervention period)<br>Authors concluded that the implemented computer-based clinical decision-support algorithm that dramatically increased Tdap vaccination uptake rate of postpartum women. The utilization of such algorithm in hospitals that have clinical decision support systems should improve rates of vaccination during postpartum period. |
| Hallas Donna et al, (2023), [44] | United States | Influenza and Tdap | Quasi-experimental intervention study. Sample n=141. | The educational intervention aimed to enable pregnant women to make evidence informed decisions about accepting vaccines for themselves during their pregnancy. All study materials were provided online. The decision to use the online format was based on the knowledge that social media impacts decision-making, especially regarding vaccination of pregnant woman and of children. The intervention was created to motivate participants to seek further information from scientific sources that were available to all study participants on the researchers' website. In this study participants who indicated that they intended to receive recommended vaccinations were treated as vaccine acceptors and served as the control group. However, if the answer was "maybe" or "no," they were included in the intervention group. | Vaccine-hesitant pregnant women, 82% had full prenatal vaccination coverage after receiving the intervention. The implemented intervention for vaccine-hesitant pregnant women was effective in shifting their status from hesitant to acceptors. |
| Elizabeth Helen Hayles et all, (2014), [45] | Australia | Tdap | Quasi-experimental intervention study. Sample n=1080. | The intervention aimed to evaluate the role of message-framing vs. standard health information in the promotion of Tdap vaccination among postpartum women. Postnatal women self-completed a pertussis knowledge and attitudes survey. Then, pertussis-susceptible (no pertussis vaccine ≤10 | Among susceptible mothers, 70%were vaccinated post-intervention. No difference in vaccination rates were similar between 'gain', 'loss' or 'control' pamphlets (69.1% vs. 71.8% vs. 68.8%). Message-framing of the |

| | | | |
|---|---|---|---|
| | | years) participants were assigned to receive a gain-framed, loss-framed pamphlet or control (Government Pertussis factsheet). Finally, participants were offered a Tdap vaccine and completed a post-questionnaire prior discharge. | educational intervention did not significantly increase vaccine uptake. Intention to be vaccinated, perceived vaccine benefits and having received a vaccine recommendation were independent predictors of vaccine uptake. At discharge, overall pertussis vaccine coverage had increased from 23% to 77%, the 'trusted' environment with minimal access barriers had increased baseline pertussis vaccine coverage from 23% to 77%. |
